# Danhong injection reduces vascular remodeling and up-regulates the Kallikrein-kinin system in spontaneously hypertensive rats

**DOI:** 10.1038/s41598-017-04661-1

**Published:** 2017-06-27

**Authors:** Xiaohu Yang, John Orgah, Dandan Wang, Guanwei Fan, Hu Jingyang, Jihong Han, Gangjian Qin, Xiumei Gao, Yan Zhu

**Affiliations:** 1 Tianjin State Key Laboratory of Modern Chinese Medicine, Tianjin University of Traditional Chinese Medicine, 312 Anshanxi Road, Nankai District, Tianjin 300193 P. R. China; 2 Research and Development Center of TCM, Tianjin International Joint Academy of Biotechnology & Medicine, 220 Dongting Road, TEDA, Tianjin 300457 P. R. China; 3 College of Life Sciences, Nankai University, Tianjin, 300193 P. R. China; 4 Feinberg Cardiovascular Research Institute, Department of Medicine, Northwestern University Feinberg School of Medicine, Chicago, IL USA; 5 Molecular Cardiology Research Institute, Tufts Medical Center, 750 Washington St, Boston, MA 02111 USA; 6 Department of Pharmacy, Zhejiang Hospital, 12 Lingyin Road, Xihu District, Hangzhou, Zhejiang 310013 P. R. China

## Abstract

Although Danhong injection (DHI) is one of the most prescribed cardiovascular medicines in China, its therapeutic indications and mechanisms remain partially defined. We now identify molecular targets of DHI in resistance vasculatures and demonstrate its role in vascular function and blood pressure (BP) regulation. BP was determined in DHI, Losartan, and placebo- treated Spontaneously Hypertensive Rats (SHR) by both noninvasive and invasive measurements. Vasorelaxation was examined both in conduit and resistance vasculature by *ex vivo* aortic rings. Microarray analysis was performed and gene expression changes were verified by RT-qPCR and ELISA. Diastolic, systolic and mean BPs were significantly lower in DHI-treated SHR than controls by both tail-cuff and invasive BP measurements. In *ex vivo* rings, aortic and mesenteric vessels from SHR treated with DHI exhibited significantly greater acetylcholine-mediated relaxation. Among the 282 genes that are differentially expressed in microarray analysis, DHI treatment up-regulated the expression of kallikrein and plasma kallikrein B genes. DHI also significantly increased serum kallikrein content in SHR. Treatment with DHI significantly increased the ratio of aortic lumen to outer diameter. Therefore, the reduction of vascular remodeling and the up-regulation of Kallikrein-kinin system contribute, at least in part, to the antihypertensive effect of DHI in SHR.

## Introduction

Hypertension is a major public health issue and a leading cause of premature death in China^1^. Approximately 1 billion people worldwide have high blood pressure (BP), and recent results of a large prospective cohort study of Chinese population suggest that out of the 500,223 people recruited, there were approximately 162,572 people with hypertension^2^. That translates to one in three adults who suffer from high BP. Despite over $19 billion in worldwide sales of antihypertensive agents, a considerable number of patients remain resistant to treatment. Novel treatment strategies are required to address this unmet medical need. As a major cardio-cerebrovascular risk factor, hypertension causes constriction of the microvasculature, resulting in the dysfunction of target organs such as the heart, brain and kidney^3^. Because the microcirculation provides the vast majority of systemic resistance to flow, as well as virtually all the oxygen and nutrient exchange^4^, alterations in microcirculatory function and structure are of great interest in the syndrome of hypertension and its target organ consequence^5, 6^. Microvascular abnormalities during hypertension increase vascular sensitivity, vessel wall thickness and loss of capillaries^7^.

The etiology of elevated BP cannot be determined in the vast majority of individuals with essential hypertension and many systems have been shown to contribute to BP homeostasis, including the vasculature, the central and sympathetic nervous systems, and the kidney, along with their various hormonal regulators. Multiple pathways such as the renin-angiotensin aldosterone system (RAAS), NO/cGMP^8, 9^ and Kallikrein-kinin system (KKS) are considered as the major BP-regulating mechanisms^10–12^. Normal levels of kallikrein are required in the maintenance of BP since tissue kallikrein levels are reduced in humans and/or in animal models with hypertension^11, 13^. Genetic models of kallikrein deficiency further support the role of kallikrein in BP regulation^14^. Additionally, up-regulation of kallikrein also has a critical role in vascular protection, especially in prophylaxis of vascular smooth muscle cell (VSMC) proliferation, vascular wall thickness and vascular remodeling^15–19^.

Quite a large number of clinical evidence suggested that multiple drug combinations produce BP reductions superior to single drug use and may help difficult-to-treat patients to achieve BP control^20^. Accordingly, herbal medicine, a natural multi-component medicine, has long been used in treating cardiovascular disease such as hypertension^8^. Although many herbal-derived antihypertensive medicines are shown to be safe and effective, complex chemical nature have limited in-depth investigation of their action mechanisms^8, 21, 22^.

Microarray technology has been successfully applied to elucidate the mechanism associated with complex therapeutic effects of herbal medicine^23^. Analyzing the alterations of gene expression profiles after treatment by herbal medicine in *vitro/vivo* may help elucidate their mechanisms of action^24, 25^.

Danhong injection (DHI), a traditional Chinese Materia Medica standardized product extracted from Radix *Salviae miltiorrhizae* (Danshen) and *Flos Carthami tinctorii* (Honghua)^26^, was approved by State Food and Drug Administration of China (Permission Number Z20026866). It was formulated based on the traditional Chinese medicine (TCM) theory of “activating and promoting blood circulations to dissipate blood stasis and dredge collaterals”^27^.

To our knowledge, DHI is among the best chemically characterized complex herbal medicine. We have previously characterized the major constituents of DHI by ultra-performance liquid chromatography (UPLC)^28^ and proton nuclear magnetic resonance (^1 ^H NMR)^29^. Recently, Zhang *et al*. have separated and identified further constituents in DHI by UPLC^30^.

DHI has been widely used in Chinese hospitals and clinics with proven efficacy and safety^31^ for the treatment and prophylaxis of various cardio-cerebrovascular diseases, such as coronary heart disease, atherosclerosis, and ischemia-reperfusion injury^32–34^. Recently published evidence suggests that DHI exert anti-cardiac hypertrophic effect by regulating p38 and NF-κb pathway^35^, ameliorates cardiac dysfunction and ventricular remodeling after myocardial infarction^36^, and relaxed norepinephrine-induced vasoconstriction via inhibition of the intracellular calcium ions (Ca^2+^) or via a PGI_2_-mediated pathway^37, 38^. However, its role on BP regulation and vascular protection has not been explored.

The present study aimed to investigate (1) the potential role of DHI on BP regulation and vascular remodeling in spontaneous hypertensive rat (SHR), (2) the ability of DHI in restoring endothelial dysfunction in both conduit (aortic) and resistance (mesenteric) vessels of SHR rats, and (3) the underlying mechanisms and signaling pathways of the microvascular activities by DHI.

## Results

### DHI Lowered Blood Pressure in SHR

**Figure 1:**
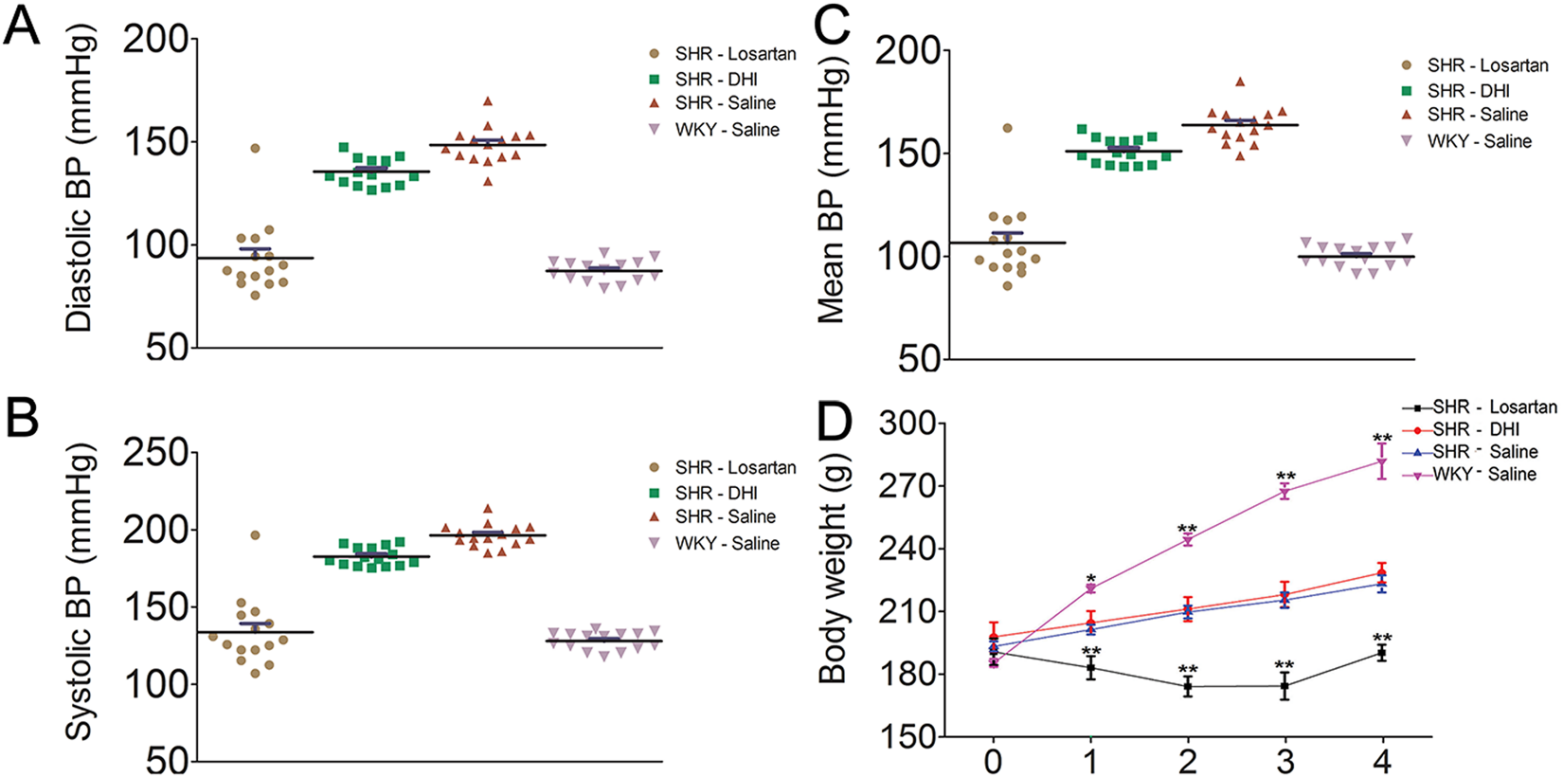
Effects of DHI on noninvasive blood pressure in SHR. Panel A–C are representative data plots from (DBP, SBP, and MBP measurements, respectively) using noninvasive tail-cuff method in conscious rats. Panel D represent body weight in (gram) measured with the small animal weighing scale. Compared with saline control, DHI-treated SHR showed no significant difference (p > 0.05 vs. saline group) in weight over the 4 weeks of routine treatment. Data are expressed as mean ± SEM, n = 4.

We first evaluated the effect of DHI on BP regulation in SHR and then compared the result with that of negative control group (SHR + Saline), taking Losartan as positive control drug. As expected, Losartan dramatically lowered diastolic, systolic and mean BP in SHR starting from day 1 till after 4 weeks (from 148.7 ± 4.2 to 107.3 ± 10.1 mmHg, p < 0.05; 194.3 ± 8.9 to 144.7 ± 9.9 mmHg, p < 0.05; and 163.8 ± 8.3 to 119.4 ± 9.9 mmHg, p < 0.05, respectively. Supplemental Materials Figure S1A–C, n = 4). Although treatment with DHI had no such immediate and aggressive effect on BP reduction in SHR as observed with Losartan, the diastolic, systolic and mean BP were all significantly decreased compared to those of saline controls within 7 days till 4 weeks (from 147.9 ± 7.9 to 127.7 ± 4.9 mmHg, 194.3 ± 8.9 to 177.3 ± 2.2 mmHg, 163.8 ± 8.3 to 143.8 ± 6.9 mmHg, respectively, p < 0.05, n = 4, Fig. 1A–C). This mild BP lowering effect of DHI was apparently less traumatic since it did not cause a significant weight loss (p > 0.05 vs. saline group, Fig. 1D), and possibly avoided detrimental organ damage in rats caused by acute reduction of BP using a BP lowering drug as previously reported^39^.

**Figure 2:**
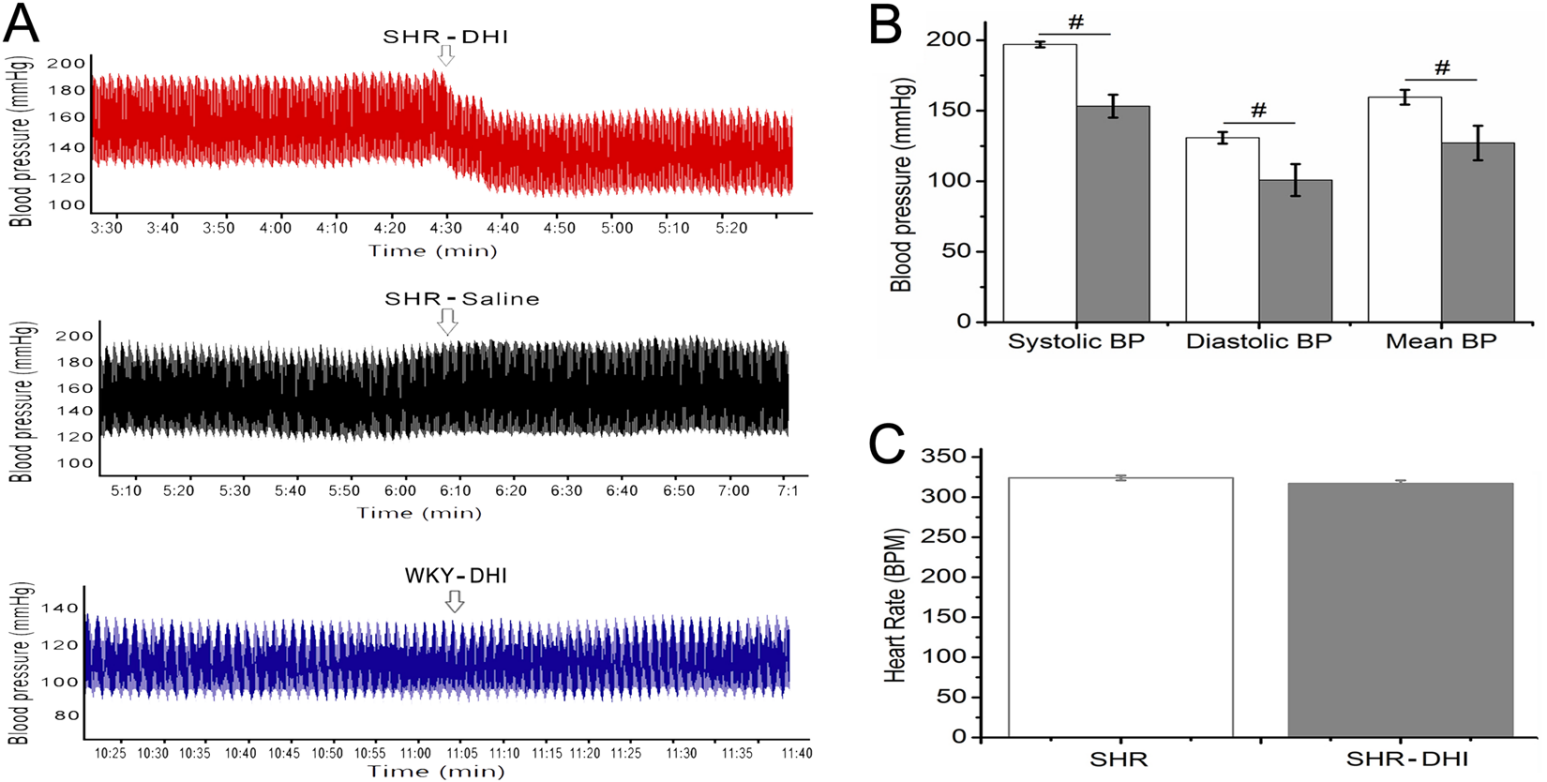
Effects of DHI on invasive blood pressure in SHR. Invasive BP were measured in urethane anaesthetised rats after treatment with DHI. A heparinized saline, 100 IU/mL was filled in the transducer in clean polyethylene catheter cannulated to the ventricle through the left common carotid artery. DHI decreased BP in SHR (p < 0.01, Fig. 2A [SHR (DHI)] and 2B, n = 3) but did not change the heart rate (p > 0.05, Fig. 2C). DHI did not change BP in normotensive WKY rats (p > 0.05, Fig. 2A [WKY (Saline)]).

In addition to noninvasive BP records, invasive BP monitoring was performed in anaesthetized rats which confirmed that DHI smoothly and effectively decreased BP (p < 0.01, n = 3, Fig. 2A, top panel [SHR (DHI)] and 2B) but did not change the heart rate (p > 0.05, Fig. 2C) in SHR. Moreover, DHI did not change BP in normotensive WKY rats (Fig. 2A, lower panel [WKY DHI]).

### DHI Improved Endothelial Dysfunction in SHR

**Figure 3:**
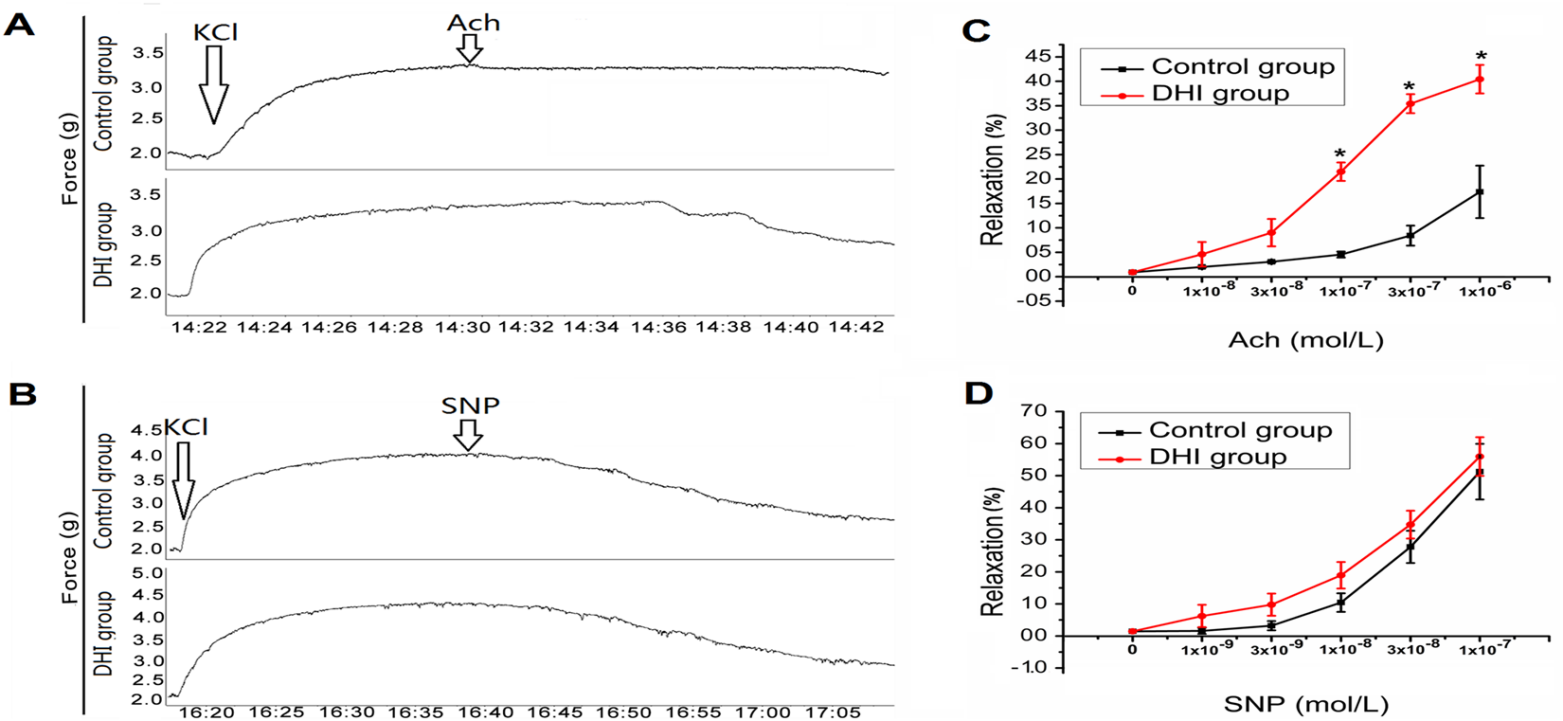
Ach and SNP-mediated vasorelaxation in KCl- precontracted thoracic aortas isolated from DHI-treated SHR. (**A**,**B**) Raw trace of Ach and SNP-treated aorta and (**C**,**D**) Quantification of the data in (**A** and **B**) respectively. The rate of Ach and SNP-induced relaxation was calculated with KCl (60 mM)-induced contraction set to 0. (**C**) DHI treatment significantly (p < 0.01) relaxed aorta in response to Ach stimulation compared with control group in SHR. (**D**) There was no significant difference in response to SNP stimulation between DHI treatment group and control group in SHRs’ aorta. All data are expressed as mean ± SEM, n = 5.

**Figure 4:**
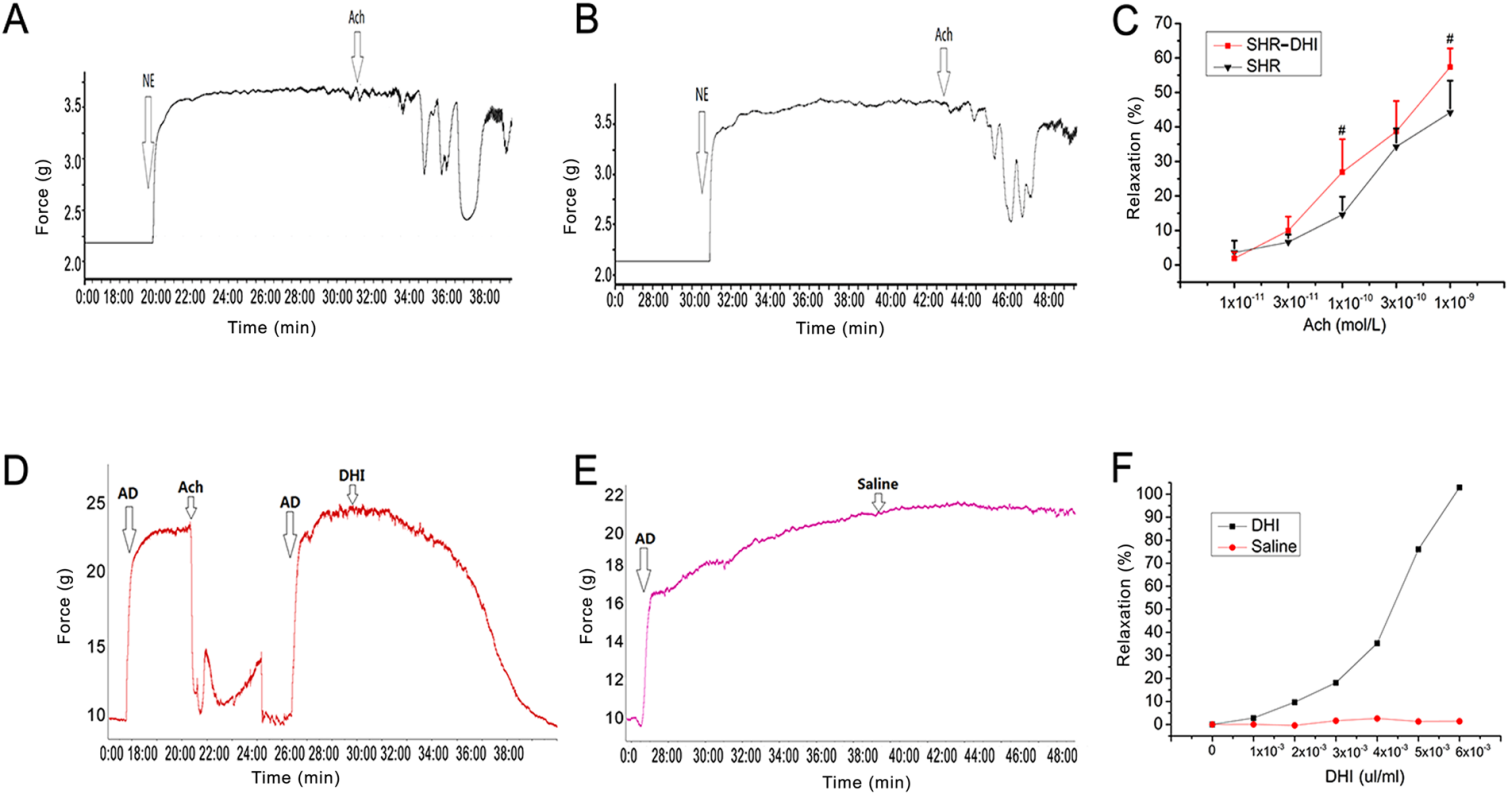
*In vivo* and *ex vivo* effects of DHI on mesenteric arteries from SHR and wild-type rats. (**A**,**B**) Raw traces of relaxation of NE-precontracted MAs from SHR in response to ACh (DHI vs. Saline treatment). (**C**) Quantitation of data in A and B expressed as % relaxation in response to increased Ach concentrations. (**D**,**E**) Raw traces of relaxation in response to ACh (DHI vs. Saline treatment) on AD pre-contracted mesenteric arteries. (**F**) Quantitation of data in (**D** and **E**) expressed as % relaxation in response to increased DHI concentrations. The rate of Ach or DHI-induced relaxation was calculated with NE (10 mM) or AD (10 mM) contraction set as 100%. (**A** and **B**) Mesenteric arteries from DHI-treated SHR animals for three days significantly relaxed more than that of saline control in response to increasing doses of ACh, p < 0.01. (**D** and **E**) DHI treatment also caused a direct endothelial-dependent vasorelaxation in AD pre-contracted mesenteric arteries from normal (WKY) rats, p < 0.01. All data are expressed as mean ± SEM, n = 3.

Since we have previously reported that DHI enhanced acute vasorelaxation in wild-type Sprague Dawley (SD) rats^38^, we conducted the same *ex vivo* vascular ring assay using thoracic aortas isolated from SHR. After one week of DHI treatment, percentage relaxation rate (%) of thoracic aortas in response to Ach (1 × 10^−8^ to 1 × 10^−6^ mol/L) was significantly improved (from 0.16 ± 0.054 to 0.39 ± 0.029, p < 0.01, n = 5, Fig. 3A and C) whereas relaxation rate (%) in response to sodium nitroprusside (SNP) remained the same (1 × 10^−9^ to 1 × 10^−7^ mol/L, p > 0.05, n = 5, Fig. 3B and D). As expected, SHR were deficient in endothelium-dependent vasorelaxation^40–42^. Because resistance vessels (mesenteric vessels) are more critical for BP control, we then conducted further vasorelaxation assay using mesenteric vessels isolated from DHI-treated SHR. After three days of DHI treatment, mesenteric arteries in SHR showed an enhanced relaxation (from 0.44 ± 0.12 to 0.58 ± 0.05, p < 0.01, n = 3) in response to Ach (1 × 10^−11^ to 1 × 10^−9^ mol/L, Fig. 4A and C) compared to controls (Fig. 4B and C). Finally, DHI caused a direct dose-dependent relaxation of isolated mesenteric vessels (Fig. 4D and F) compared to the controls (Fig. 4E and F).

### DHI Reversed Vascular Remodeling in SHR

**Figure 5:**
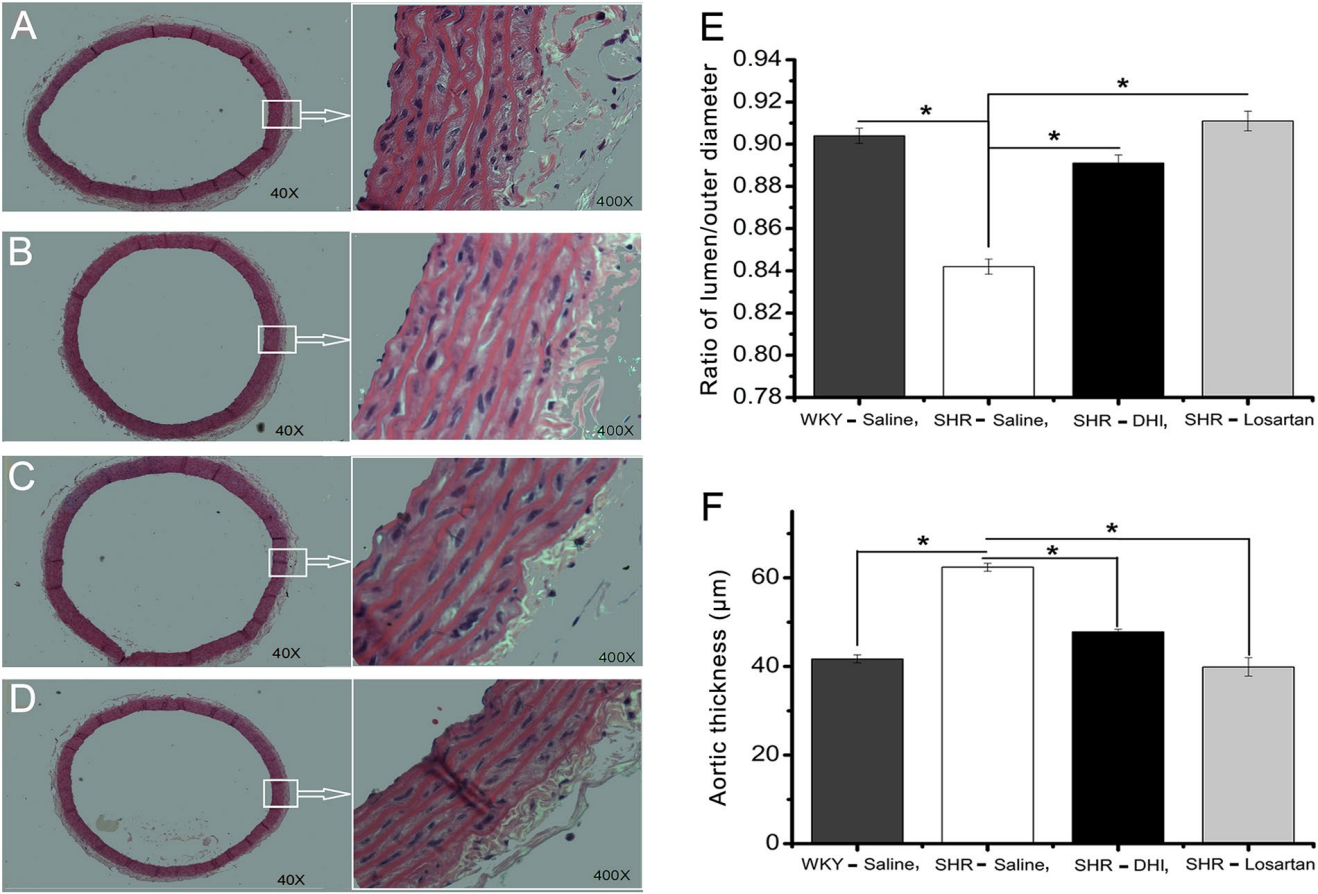
Effect of DHI on vascular remodeling in SHR. Histology of H&E stain of thoracic aorta at x40 and x400 (**A**–**D**) are shown. Summarized ratio of the inner to outer diameter of the thoracic aortas from the 4 groups of rats are quantified and presented in (**E** and **F**). (**A**) Saline treated WKY rats; B: Saline treated SHR rats; (**C**) SHR rats treated DHI; (**D**) SHR treated Losartan. Values represent mean ± SEM (n = 4 per group; p < 0.01 vs. SHR treated DHI).

Aortic thickness resulted in the reduction of their lumens. Compared with that of WKY rats, the aortas from SHR showed a significant decrease in the ratio of lumen/outer diameter (from 0.904 ± 0.007 to 0.842 ± 0.003, p < 0.05, n = 4, Fig. 5A,B and E). As expected, Losartan increased the ratio of lumen/outer diameter (Fig. 5B,D and E). Similarly, DHI treatment significantly increased the ratio of lumen/outer diameter (from 0.842 ± 0.003 to 0.891 ± 0.004, p < 0.05, n = 4, Fig. 5B, C and E). The VSMC layer of DHI-treated SHR was thinner than that of their control counterpart (from 62.4 ± 0.9 to 47.8 ± 0.6 μm, p < 0.05, n = 4, Fig. 5B [x400], 5C [x400] and 5F).

### Identification of Differentially Expressed Genes from Microarray and RT-PCR validation

In order to identify the molecular mechanisms of DHI effect on vascular relaxation and remodeling, we compared gene expression profiling between DHI-treated and control-treated SHR using the Rat **Genome-**230 2.0 microarray (31,000 genes represented). Mesenteric vessels from four independent animals per group were individually analyzed (Supplemental Figure S2A) and the results indicates that a total of 282 genes (195 up-regulation and 87 down-regulation, Supplemental Figure S2B and C, details in Supplemental Materials Table S1) were altered when a cutoff of t-test (p < 0.05) and fold changes (>2) was applied.

**Figure 6:**
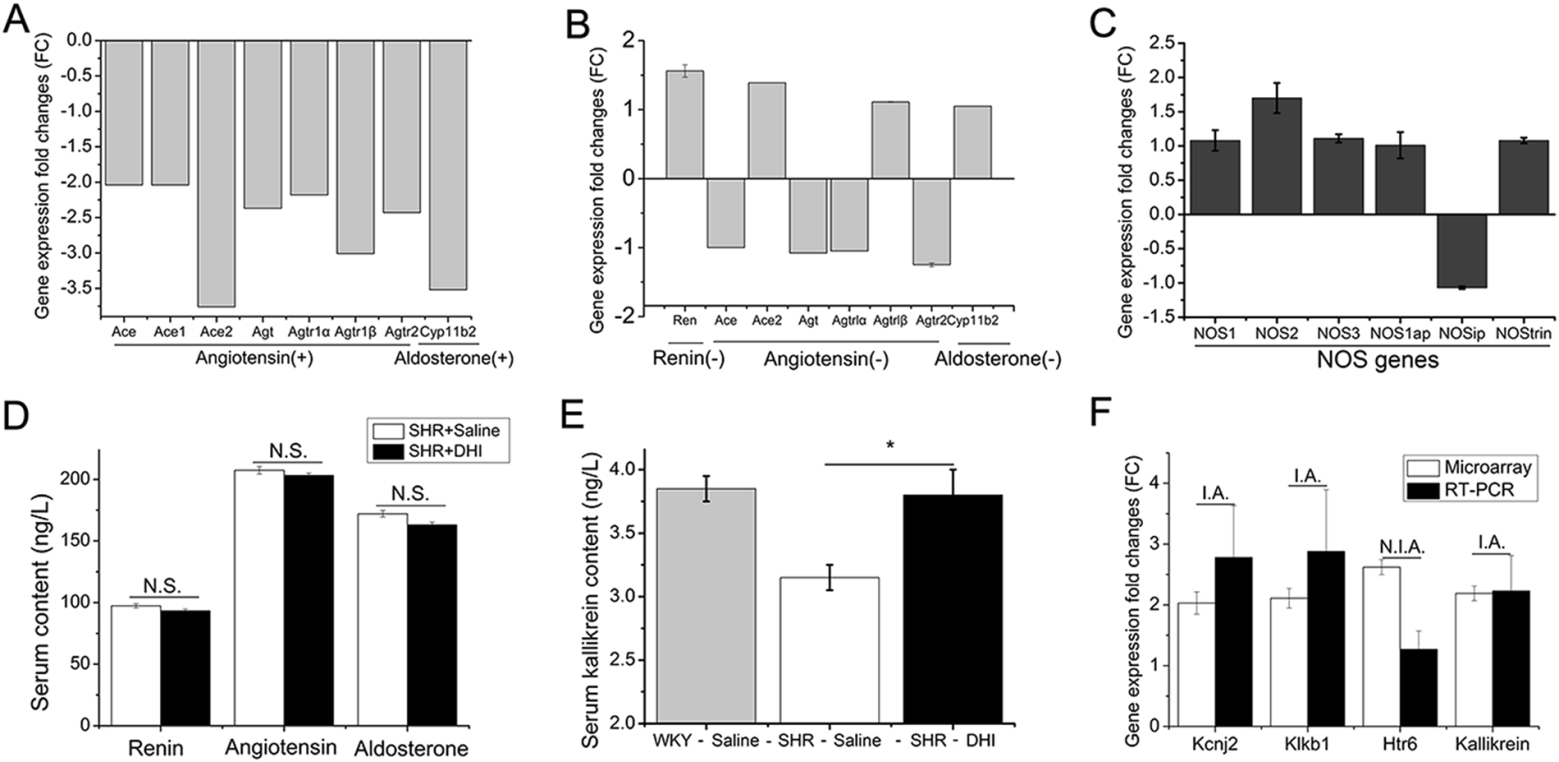
Gene expression changes by Microarray analysis and subsequent confirmation in SHR mesenteric vessels. (**A**) Microarray confirmation of down-regulation of RAAS genes by Losartan. (**B** and **C**) DHI did not alter RAAS and NOS genes in microarray profiling, respectively. (**D**) Serum renin, angiotensin and aldosterone contents were determined and no significant difference (N.S.) was detected between DHI treatment and the control groups. (**E**) Effect of DHI on serum kallikrein content determined by ELISA assay (*p < 0.05). (**F**) Other hypertension-associated genes that are differentially regulated by DHI in microarray and their confirmation by RT-PCR. RT-PCR gene expressions of Kcnj2, Klkb1, and kallikrein were in accordance (I.A) with microarray data whereas Htr6 gene expression was not in accordance (N.I.A.) with that obtained by microarrays. All data are expressed as mean ± SEM, n = 4.

The microarray data was validated as Losartan significantly down-regulated angiotensin/aldosterone genes in SHR (Fig. 6A). However, DHI did not alter these angiotensin/aldosterone genes (Fig. 6B), nor the NOS genes (Fig. 6C) in SHR. On the other hand, DHI did alter several genes known to be associated with BP-regulation and hypertension, including kallikrein, plasma kallikrein B1 (Klkb1), 5-hydroxytryptamine (serotonin) receptor 6 (Htr6) and potassium inwardly-rectifying channel, subfamily J, member 2 (Kcnj2) and their differential expressions were validated by RT-PCR from the same RNA source (Fig. 6F). Of particular interest, the expression of kallikrein and Klkb1 genes were up-regulated in microarray by 2.19 ± 0.12-fold change and 2.11 ± 0.16-fold change respectively (p < 0.05, n = 4, Fig. 6F) which were confirmed by RT-PCR by 2.23 ± 0.57-fold change and 2.88 ± 1.01-fold change, respectively (p < 0.05, n = 4, Fig. 6F). As a control, von Willebrand factor (vWF), an unaltered gene in microarray, was also not altered in RT-PCR (data not shown).

### DHI Selectively Increased Serum Kallikrein Content in SHR

To further distinguish the BP-regulation pathways affected by DHI, we conducted ELISA assays for the relevant factors. While DHI did not alter the levels of renin, angiotensin and aldosterone contents (p > 0.05, n = 4, Fig. 6D), it significantly increased serum kallikrein content (from 3.15 ± 0.1 to 3.80 ± 0.2 ng/mL, p < 0.05, n = 4, Fig. 6E) in SHR compared with the controls.

## Discussion

Formulated based on the TCM theory of “activating and promoting blood circulations to dissipate blood stasis and dredge collaterals”^34^, DHI has been widely used in Chinese hospitals and clinics with proven efficacy and safety^31^ for treatment and prophylaxis of various cardio-cerebrovascular incidents, such as treatment of ischemia-reperfusion injury, atherosclerosis, acute coronary syndrome, hepatic veno-occlusive disease^32, 33, 43, 44^. Following our previous published work^38^ showing that DHI and one of its component Danshensu serve as a direct vasodilator in normal rats, the primary finding of this study is that DHI significantly lowered BP (diastolic, systolic and mean blood pressure) in SHR compared with that of control group (Fig. 1A–C). It is worth noting that while its BP-lowering effect was not as robust as Losartan, DHI did not cause adverse effect resulting in the rapid weight loss as the Losartan treatment group (Fig. 1D). Consistent with this observation, we also found that the level of myoglobin (Mb), a biomarker for kidney injury, was much higher in Losartan group compared to those of DHI and control groups (data not shown). The milder and smoother effect of DHI in lowering BP compared with Losartan (Fig. 1A–C) indicated that DHI may avoid some of the known adverse effects of antihypertensive drugs^8, 39, 45^. Furthermore, the dual effects of direct vasorelaxation and long-term vascular remodeling by DHI support the effectiveness of multi-targeting strategy in blood pressure control, an advantage of Traditional Chinese Medicines with multicomponent combinations.

DHI is formulated as an injection using aqueous extracts from *Radix Salviae miltiorrhizae* (Danshen) and *Flos Carthami tinctorii* (Honghua) and has been shown significantly to vasodilate isolated arterial rings in rats^38^. Several studies have indicated that various extracts and components from either Danshen or Honghua may have anti-hypertension activities^46–48^. Pei-He Nie *et al*.^46^ and David D. Kim *et al*.^48^ reported that HSYA (a hydrophilic compound from Honghua) and tanshinone IIa (a lipophilic compound from Danshen) had an antihypertensive effect. However, our recent chemical composition analysis of DHI^28^ showed that neither HSYA nor tanshinone IIa were detectable. Therefore, the antihypertensive effect of DHI in our study could not be attributable to HSYA and tanshinone IIa. Other studies also found that antihypertensive effects of Danshen and Danshensu (a water-soluble compound from Danshen) were mediated by the inhibition of angiotensin conversion enzyme (ACE) and the activation of NOS/NO, respectively^9, 47^. Although our previous study showed that Danshensu was a major component of DHI involved in endothelium-dependent vasorelaxation in wild-type SD rats^38^, our current microarray analyses of resistant vessels from SHR demonstrated that ACE and NOS gene expression were not altered by DHI (Fig. 6B and C). These findings provided a clear distinction of antihypertensive effects of DHI from isolated compounds and extracts of Danshen and Honghua reported previously.

Since DHI is an herbal extract-derived medicine, its antihypertensive effect could be contributed by multiple components through a variety of mechanisms. Therefore, we performed a microarray analysis of the genes in resistance vessels to discover the potential genes regulated by DHI and to investigate the mechanistic insights of its BP-lowering effects. The microarray data for 8 samples were firstly analyzed by hierarchical clustering analysis for a quality evaluation and a comparison of the treatment effects. The biological replicates in each treatment group showed high reproducibility judged by heat-map analysis (Supplemental Figure S2A). Our results indicate that in micro-vessels of SHR, DHI altered multiple pathways such as insulin signaling pathway, glutathione, mTOR signaling pathway (See details in Supplemental Materials Table S3).

Microarray data analyses indicated that in contrast to that of Losartan (Fig. 6A), DHI did not alter the expression of genes such as renin, angiotensin and aldosterone (RAAS, Fig. 6B), suggesting that the DHI pathway for reducing BP was not mediated by RAAS. On the other hand, we found several differentially expressed genes that were related to BP regulation, especially kallikrein gene that was significantly overexpressed (Fig. 6F).

RAAS and KKS are two major circulating endocrine systems in the regulation of BP^10, 49^. Cross-talks exist between the two systems and their levels of expression showed a complex pattern: both RAAS and KKS would be up-regulated in some circumstances, whereas in other circumstances they respond to opposite direction, expressed as an activated KKS and a depressed RAAS^50^. As the cross-talks between the RAAS and KKS play a critical role in regulation of BP^51^, we further investigated if DHI affected one or both of the pathways. Indeed, our ELISA tests of serum renin, angiotensin, aldosterone and kallikrein (Fig. 6D and E) were in excellent accord with the gene expression data (Fig. 6B and F). As a main component of KKS, Kallikrein plays a crucial role in regulation of BP and thus a target for antihypertensive drugs^14, 52, 53^. In our study, results of ELISA analysis and microarray data indicated consistency with several recent studies^14, 54^, kallikrein gene overexpression in mesenteric micro-vessels increased the serum kallikrein content. Therefore, our results seem to support a differential pattern of RAAS and KKS by DHI and the BP-lowering effect of DHI is preferentially triggered by KKS mediated by kallikrein gene overexpression.

The onset and development of hypertension is often accompanied by vascular structure and function abnormalities such as endothelial dysfunction and the hypertrophy, hyperplasia and connective tissue increase of the blood vessels^55, 56^. In the current study, we found that DHI increased endothelium-dependent relaxation (23 ± 3%, p < 0.01, Fig. 3C) but not endothelium-independent relaxation of aortas from SHR (p > 0.05, Fig. 3D). This finding is consistent with our previous study using wild-type SD rats that DHI and one of its active components, Danshensu, promoted vasorelaxation *in vivo* (2 day-treatment) and *ex vivo* by a COX/PGI_2_-mediated pathway^38^. Interestingly, we have shown recently that a longer exposure (4 weeks) of DHI in SD rats further increased endothelium-dependent relaxation (19% ± 2%, p < 0.01, data not shown) compared to the acute exposure (2 days) reported previously^38^. Consistent with these findings, overexpression of tissue kallikrein gene promoted KKS activation, which enhances prostacyclin2 and nitric oxide biosynthesis, both of them facilitated the regulation of vascular responses^18, 57^.

Additionally, increased vascular wall thickness is a common structural feature of hypertensive resistance vessels and conduit arteries such as the aorta^22, 58^. Chronic hypertension changes the dimensions and properties of arterial wall and these alterations may affect arterial mechanics. Therefore, vascular remodeling, a pathological process involving VSMCs proliferation, migration, hypertrophy, vascular compliance reduction and narrowing of the vessels lumen, cause a major damage of hypertension^59^. Study of Bo Wang *et al*.^22^ demonstrated that Qin-Jiang-Ya-Tang (TCM) could reverse thoracic aortas remodeling in SHR. In our present study, DHI significantly decreased SHR aortic thickness compared with that of control group (Fig. 5E and F). Numerous studies have demonstrated that either tissue or serum kallikrein overexpression decreases vascular wall thickness, inhibited VSMCs proliferation and reversed vascular remodeling^60, 61^, and our results indicated that the long-term effect of DHI on vascular remodeling could also be mediated by the KKS via kallikrein up-regulation.

Finally, it is worth noticing that the vast majority of over 200 differentially expressed genes by DHI treatment in the microarray study (supplemental material Table S2) remain unexplored. Future studies using combined approaches of bioinformatics and molecular biology may further elucidate DHI’s molecular targets and therapeutic potentials in hypertensive resistance vessels. Also, to further validate the proposed anti-hypertensive mechanism of DHI via KKS modulation, future study need to focus on directions to show that blocking KKS by inhibitors or using gene knockout mice would prevent DHI mediated BP lowing, vasorelaxation and remodeling. Since we have only investigated the whole DHI, another required research direction is to define the precise active components that are responsible for the cardiovascular benefits as a complex herbal medicine.

In conclusion, Chinese medicine DHI effectively lowers blood pressure without causing significant adverse effect. This effect is attributable to DHI’s ability of enhanced vasorelaxation and reduced vascular remodeling. The antihypertensive action and vascular protection by DHI in SHR model are at least in part mediated by KKS via up-regulation of kallikrein gene expression.

## Materials and Methods

### Reagents

DHI was supplied by HEZE BUCHANG PHARMACEUTICAL CO., LTD. Losartan was purchased from the Yangtze River Pharmaceutical Group Sichuan sea pureed pharmaceutical Co., LTD (Dujiangyan, Sichuan, China). Saline was purchased from China Otsuka pharmaceutical co., LTD (Tianjin, China). Adrenaline (AD) was purchased from (Shanghai Harvest Pharmaceutical Co., Ltd, China). All other reagents were purchased from Sigma-Aldrich (St Louis, MO). Losartan was dissolved and diluted by saline. In *vivo/vitro* DHI doses were according to our previous report^38^.

### Animals and Treatment

Adult (12 week-old) male SHR and age-matched male normotensive Wistar-Kyoto (WKY) rats, weighting about 200 g, were purchased from Vital River Experimental Animal Technology Co., LTD (Beijing, China). Rats were intraperitoneally injected with DHI (2.5 mL/kg/day), Losartan (20 mg/kg/day), and/or saline (2.5 mL/kg/day) for 4 weeks. They were maintained in a temperature-controlled room (25 °C ± 1) under a cycle of 12 hours of light (beginning at 9:00 A.M.). All rats were given water and fed with standard chow. All animal care and operation procedures were in strict accord with the China Laboratory Animal Use Regulations and were approved by the Institutional Animal Care and Use Committee at Tianjin International Joint Academy of Biotechnology and Medicine (TJAB-JY-2011-002), Tianjin, China.

After 4 weeks of treatment, animals were anesthetized using intraperitoneal injection of 10% Chloralic Hydras. Serum from blood was collected post-anesthesia and stored at −20 °C for ELISA assay. Thoracic aortas and mesenteric micro-vessels were harvested. Aortas were placed in 10% formalin for hematoxylin and eosin (H&E) staining, and mesenteric micro-vessels were frozen in liquid nitrogen for microarray analysis and real time RT-PCR verification.

### BP Measurements

Conscious noninvasive tail-cuff BP measurements were performed every two days for 4 weeks after the initiation of the drug treatments using an 8-channel CODA noninvasive BP acquisition system (Kent Scientific Corporation, CT, USA) following the manufacturer’s protocol. Measurements were recorded after 7 days of training. Systolic, diastolic and mean BP as well as heart rate was recorded. For BP recording, eight separate measurements were obtained and averaged for each rat.

Invasive Blood pressure and heart rate where measured using ADInstruments PowerLab 8/30 connected to bridge Amp, ML 221, invasive blood pressure (BP) was recorded alongside heart rate after rats were treated with DHI and changes in BP was observed. Briefly, Animals were anesthetized with urethane and surgical manipulation was carried out on the animals. A heparinized saline, 100 IU/mL was filled in the transducer in clean polyethylene catheter cannulated to the ventricle through the left common carotid artery to prevent blood clotting. BP and HR were continuously recorded for 3 min each as waveform curve and the software calculated their values.

### Aortic Ring Assay

The aortic ring assay was performed as previous described^62^. Briefly, segments of aortas (3 mm) were dissected out and placed in organ baths containing 20 mL Krebs buffer (mM: NaCl 118, KCl 4.7, MgSO_4_ 1.2, KH_2_PO_4_ 1.2, NaHCO_3_ 25, CaCl_2_ 2.5, glucose 5.5); optical resting tension was determined in baseline experiments performed before treatment with the compounds under investigation. The rings were gradually stretched to an optimum preload of 2.0 g of force, determined in previous experiments in this laboratory^38^. Vessels were sub-maximally pre-contracted with KCl (60 mM), the endothelial function was evaluated by vascular relaxation in response to acetylcholine (Ach) and the vascular smooth muscle function was evaluated by vascular relaxation in response to sodium nitroprusside (SNP). Isometric forces were recorded with force transducers connected to a PowerLab/870 Eight-channel 100 kHz A/D converter (AD Instruments, Sydney, Australia).

### Isolation and Mounting of Small Mesenteric Artery Segments

Rats were killed by an overdose of isoflurane and the mesentery was removed and placed in cold Krebs-Ringer buffer (KRB) with the following composition (in mM): 118.5 NaCl, 4.7 KCl, 2.5 CaCl2, 1.2 MgSO4, 1.2 KH2PO4, 25.0 NaHCO3, and 5.5 D-glucose. From each rat, segments (2 mm) of the second-order branches of the superior mesenteric artery (MA) was carefully dissected and mounted on a wire-myograph (model 620 M; Danish Myotechnology, Aarhus, Denmark) for the recording of isometric force development. MAs were incubated for 30 min in KRB with continuous aeration in 95% O_2_/5% CO_2_ and were maintained at 37 °C. MAs were passively stretched according to a procedure first described by Halpern and Mulvany^63^. In brief, MAs were stretched stepwise to a passive wall tension of 90% of the internal circumference achieved when they were exposed to a passive tension yielding a transmural pressure of 100 mmHg. At this passive wall tension, MAs were contracted with high K^+^ KRB (60 mmol/L KCl in KRB solution; replacing equimolar NaCl with KCl), thus generating a stable contraction that reached a plateau after 10–15 min. This active wall tension was set to a 100% contraction level.

### RNA Extraction

Total RNAs were extracted from rat mesenteric micro-vessels using RNeasy Mini Kit (QIAGEN, Valencia, CA), following the manufacturer’s protocol. RNA concentrations were measured by ultraviolet spectrophotometer and denaturing gel electrophoresis. All raw RNA samples were purified and concentration adjusted to 50 ng/μL. The RNA samples were stored in liquid nitrogen before further processing for microarray analysis and real time RT-PCR verification.

### Microarray Analysis

Only the RNAs with RNA integrity numbers (RINs) greater than 7.0 and a 28SrRNA/18SrRNA ratio more than 0.7 were used for microarray experiments. Gene expression data were generated using Affymetrix Rat 230 plus 2.0 arrays (Affymetrix, Inc. USA) for 4 independent animals. Each array consists of 31,000 probe sets/genes (Seen detailed procedures for Supplemental Materials).

### ELISA Analysis

Serum from each treatment group was collected and the contents of renin, angiotensin, aldosterone and kallikrein were detected using ELISA Kit (R&D Systems, Inc. MN USA) according to the manufacturer’s instruction (Seen detailed procedures for Supplemental Materials).

### Histological Studies

Aorta specimens from each animal were fixed in 10% formalin, paraffin-embedded and sectioned at 5 μm thickness, deparaffinized and rehydrated. Histological staining was performed as previous described^64^. H&E were used to evaluate the general histology of aorta. The whole aortas with lumen and outer diameters were photographed and the mean arterial wall thickness (defined as the ratio of lumen/outer diameter) was determined by a computerized ImageJ software (National Institutes of Health, USA).

### Statistical Analysis

All values are presented as means ± SEM with “n” being the number of individual rats. Statistical analyses were performed by one-way ANOVA followed by Bonferroni multiple comparisons test (95% confidence interval) from SPSS11.5 and we used Origin 8.5.1 software (Origin Lab Ltd, USA) for data analyses. Values of p < 0.05 were considered to be statistically significant.

## Electronic supplementary material

Supplemental materials
