# Supplementary material for: Danhong injection reduces vascular remodeling and up-regulates the Kallikrein-kinin system in spontaneously hypertensive rats
Source: Sci Rep. 2017 Jun 27;7:4308. doi: 10.1038/s41598-017-04661-1 (PMC5487322; doi:10.1038/s41598-017-04661-1)
Supplement: Supplementary file 1 — Supplemental materials [file 41598_2017_4661_MOESM1_ESM.doc]

**Danhong injection reduces vascular remodeling and up-regulates the Kallikrein-kinin system in spontaneously hypertensive rats**

Xiaohu Yang, John ORGAH, Dandan Wang, Guanwei Fan, Jingyang Hu, Jihong Han, Gangjian Qin, Xiumei Gao, Yan Zhu

# Supplemental Materials

**EXPANDED MEDTHODS**

**Enzyme-linked immune sorbent assay**

ELISA assays were performed according to manufacturer’s instructions with the following modifications. Before experiment, blood plasma was spun down for 20 min at 2000-3000 rpm to avoid precipitation. Supernatant was used for renin, angiotensin, aldosterone and kallikrein Elisa assays. Samples and standards were serially diluted and incubated for 30 min at 37°C, washed with buffer 5 times, and dried. Samples were then incubated with HRP-Conjugate reagent for 30 min at 37°C, and then washed again. Chromogenic solutions were then added to each well, and further incubated in dark for 15 min at 37°C, and the reaction was terminated by stop solution. Samples absorbance were read at 450nm and calculated by standard curve fitting to the straight line regression equation.

**Microarray Procedure**

The cDNA synthesis and labeling were carried out following Affymetrix one-cycle sample preparation protocol. Under the action of reverse transcriptase, adding T7-(dT)_24_ as primer, 0.1 μg of total RNA from each sample, along with poly A spikes (labeling control), were converted to double-stranded cDNA with GeneChip One-Cycle cDNA synthesis kit (Affymetrix). After second-strand synthesis, the cDNA was purified with GeneChip sample cleanup module (Affymetrix). Biotinylated aRNAs were synthesized by in vitro transcription using the Affymetrix GeneChip 3ʹ-Amplification kit. For each sample, 15 μg biotinylated aRNA along spiked controls (bioB, bioC, bioD and cre) was hybridized to a Rat 230 plus 2.0 Array for 16 hours at 45°C. Following hybridization, arrays were washed, stained and then scanned with a Scanner 3000 7G 4C Autoloader (AffymetrixGeneChip 3000 TG System).

**Microarray Data Analysis**

Microarray data obtained for this study are Minimum Information about a Microarray Experiment (MIME) compliant. The overall data are available in the GeneBank, LocusLink and UniGene of the National Center for Biotechnology Information, respectively (<http://www.ncbi.nlm.nih.gov/>). The microarray gene expression data were imported to dChip software ([www.dchip.org](http://www.dchip.org/)) and the probe set-level expression data were summarized from probe-level data with Robust Multichip Average (RMA) by combining all 8 microarray data sets. Statistical testing and clustering analysis were conducted using dChip. For each probe set, log_2_-transformed intensity data were used in a two-sample t-test to obtain a p-value and a fold change (FC).

**Quality Assessment of Microarray Data**

Hierarchical clustering analysis combined with heat-map was applied to evaluate the overall reproducibility and variation of 4 replicates within each group and the differences between the 2 groups. The log_2_-transformed expression intensities of 31,000 probe sets/genes with RMA summarized data from 8 microarrays were used to calculate the correlation coefficients between two gene expression profiles and constitute the heat-map. The quality of microarray data generated in this study is excellent for identifying the differential gene expressions.

**Hierarchical Clustering Analysis for Quality Assessment of Microarray Data and Identification of Treatment Effects**

Hierarchical clustering analysis was used to evaluate the overall quality of the microarray data. A high correlation coefficient (colored in red in the heat-map of correlation coefficients) means that the gene expression profiles from two microarrays are absolutely similar (Figure 3A of the full text). The four replicates in each treatment group showed high pair-wise correlation in terms of log_2_ gene expression. In addition, samples treated with Danhong injection (DHI) showed different expression profiles dramatically compared with the control group. The visual observation of the clustering results indicate satisfactory reproducibility of microarray experiments for the biological replicates in each treatment group and crucial treatment effects of DHI, pledging the further analyses and elucidation of their treatment effects.

**Identification of Differential Gene Expressions**

Differential gene expressions between two sample groups were selected following the recommendations of the MAQC project [1, 2]. P-value and a fold change were calculated by comparing the treatment group with the control. The probe sets/genes with p-value greater than a pre-defined cutoff (p > 0.05) were removed and the remaining of probe sets/genes was ranked through the magnitude of fold changes (FC). Gene expressions (FC) greater than a pre-defined threshold (e.g. FC > 2.0) were considered as differential expressions as previously shown [3-5]. A p < 0.05 and FC > 2.0 were set for this study to examine the genes that are dominantly regulated by DHI. To reduce the number of false-positives, selected genes of interest were therefore validated by real time RT-PCR.

**Real-Time RT-PCR**

Real-time RT-PCR was performed to validate the microarray results. One microgram of total RNA from the same of mesenteric micro vessels was incubated with DNase I, and reverse-transcribed with oligo dT using Superscript II RT-PCR (TIANGEN). 400 nanogram of RT product was amplified by primer pairs specific for the following genes: kallikrein, Klkb1, Kcnj2, Htr6, and vWF. B_2_M gene was used as a normalizing control. The primer sequences are listed in Table 4 of Supplemental Materials. SYBR Green PCR was performed using CFX96 Real-time system (Bio-Rad, CA, USA). For all reactions, a standard amplification program was used (1 cycle of 95°C for 2 min, 44 cycles of 95°C for 30 s, 60°C for 40 s and 72°C for 40 s, 1 cycle of 95°C for 10 s). The data was calculated by melting curve and ‘Threshold Cycle’ (Ct) using CFX Manager Software.

**Legends TO ONLINE FIGURES AND TABLES**

**
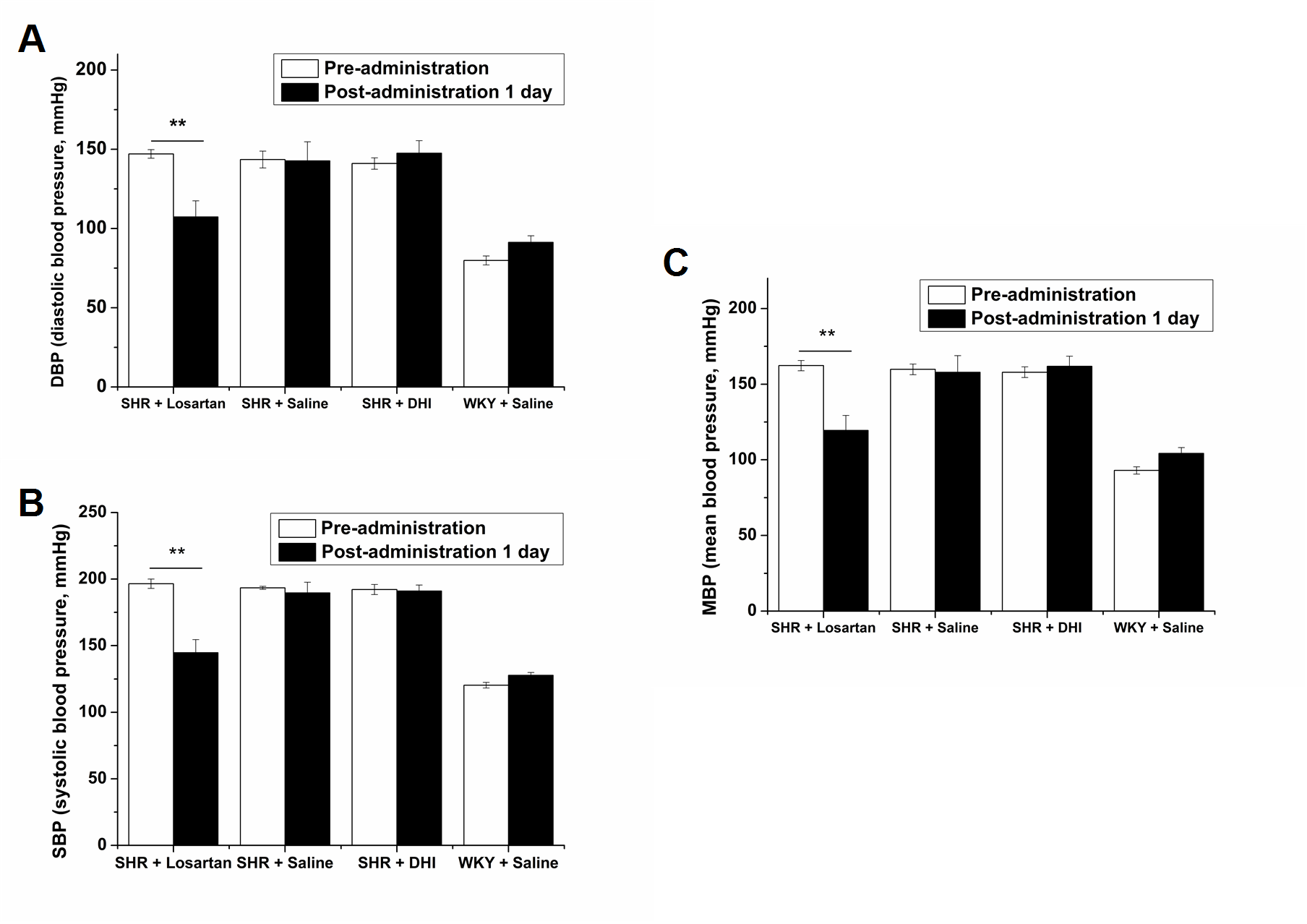
**

**Supplemental Figure S1. Short-term effect of Losartan and DHI on blood pressure in SHR** Losartan lowered DBP (from 148.7 ± 4.2, to 107.3 ± 10.1 mmHg), SBP (from 194.3 ± 8.9, to 144.7 ± 9.9 mmHg) and MBP (from 163.8 ± 8.3, to 119.4 ± 9.9 mmHg) whereas no significant difference between DHI treatment and the control was detected. A: DBP, diastolic blood pressure; B: SBP, systolic blood pressure; C: MBP, mean blood pressure. **p < 0.01, n=4.

**
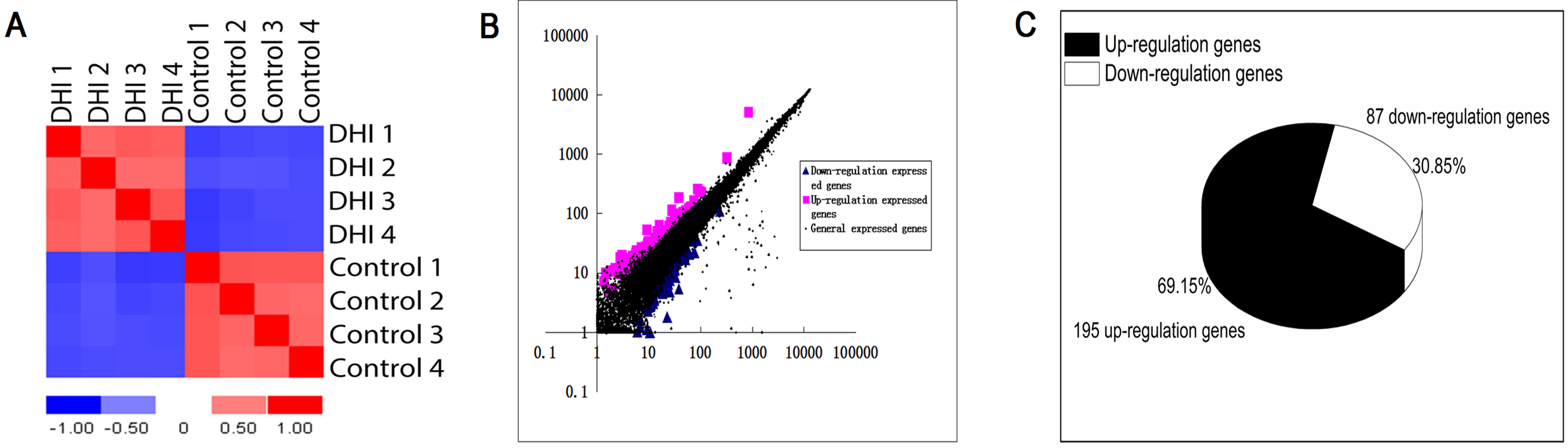
**

**Supplemental Figure S2.** Genes and pathways affected by DHI in micro-vessels and circulation of SHR. A: Heat-map of the correlation coefficients between gene expression profiles by hierarchical clustering analysis. B: Scattered plot of differentially expressed genes in DHI treatment and control groups. C: Genes that was up or down-regulated by DHI treatment when the cutoff value is set at fold changes > 2.

**Supplemental Table S1. List of differentially expressed genes by microarray data**

| Accession | Genes | Fold changes | P-value |
| --- | --- | --- | --- |
| BI289525 | Gdf1 /// Lass1: growth differentiation factor 1 /// LAG1 homolog, ceramide synthase 1 | -12.53 | 0.046996 |
| NM_133608 | Slco4a1: solute carrier organic anion transporter family, member 4a1 | -10.4 | 0.030845 |
| BF282712 | Tmem82: transmembrane protein 82 | -8.11 | 0.02474 |
| BM385351 | Syt15: synaptotagmin XV | -6.96 | 0.010325 |
| AW917556 | Rn.19456.1 | -5.87 | 0.028375 |
| BG372536 | Polk: polymerase (DNA directed) kappa | -5.41 | 0.016505 |
| AI716115 | Tmem151a: transmembrane protein 151A | -5.15 | 0.048993 |
| BM391741 | Scarf2: scavenger receptor class F, member 2 | -4.23 | 0.028923 |
| AA964809 | Rn.36146.2 | -4.2 | 0.039779 |
| BF404988 | Usp11: ubiquitin specific peptidase 11 | -4.16 | 0.041671 |
| AW529441 | LOC686841: similar to Protein EAN57 | -3.93 | 0.014822 |
| BF406285 | Pyroxd2: pyridine nucleotide-disulphide oxidoreductase domain 2 | -3.92 | 0.006345 |
| BG668764 | Spock3: sparc/osteonectin, cwcv and kazal-like domains proteoglycan (testican) 3 | -3.91 | 0.013958 |
| BF405043 | Rn.57843.1 | -3.84 | 0.032829 |
| NM_024000 | Camkv: CaM kinase-like vesicle-associated | -3.77 | 0.038548 |
| AF080594 | Vegfa: vascular endothelial growth factor A | -3.75 | 0.010551 |
| AI511059 | Trib1: tribbles homolog 1 (Drosophila) | -3.64 | 0.02059 |
| BI290256 | Stx1a: syntaxin 1A (brain) | -3.6 | 0.013546 |
| BF393691 | Rn.57742.1 | -3.59 | 0.028427 |
| BF398558 | Rn.59115.1 | -3.58 | 0.039028 |
| BF403055 | Poln: polymerase (DNA directed) nu | -3.53 | 0.005847 |
| AW919180 | Pygm: phosphorylase, glycogen, muscle | -3.45 | 0.007136 |
| AI073065 | Gpr143: G protein-coupled receptor 143 | -3.44 | 0.042965 |
| AI555848 | Rn.8304.1 | -3.38 | 0.030099 |
| AW526951 | Rn.49099.1 | -3.37 | 0.001818 |
| BE110888 | Rn.51939.1 | -3.34 | 0.022632 |
| AW142811 | Rn.17267.1 | -3.32 | 0.042565 |
| BF416348 | Rn.72167.1 | -3.3 | 0.013464 |
| BF545988 | Tmc4: transmembrane channel-like 4 | -3.3 | 0.015167 |
| BF391128 | Rn.57365.1 | -3.19 | 0.019025 |
| BI296432 | Rn.41413.1 | -3.18 | 0.047806 |
| AW535601 | Rn.47493.1 | -3.03 | 0.049362 |
| AA957632 | Rn.32930.1 | -2.92 | 0.044589 |
| BE098114 | Cip98: CASK-interacting protein CIP98 | -2.88 | 0.036609 |
| BI288963 | Rn.52153.1 | -2.84 | 0.015667 |
| BE096426 | Lrrc8e: leucine rich repeat containing 8 family, member E | -2.83 | 0.022914 |
| AI575440 | Rn.18733.1 | -2.82 | 0.015873 |
| BF392832 | Rn.61282.1 | -2.81 | 0.030797 |
| AA964856 | Rn.43928.1 | -2.79 | 0.036329 |
| BF416300 | Rn.62980.1 | -2.78 | 0.013931 |
| AI044428 | Stra6: stimulated by retinoic acid gene 6 | -2.74 | 0.029179 |
| BF419551 | RGD1559885: similar to hypothetical gene supported by BC063892 | -2.69 | 0.04122 |
| AI639437 | Rn.43702.1 | -2.67 | 0.035934 |
| AI547542 | LOC678978 /// Ptpn4: hypothetical protein LOC678978 /// protein tyrosine phosphatase, non-receptor type 4 | -2.64 | 0.034437 |
| AI231356 | Rn.8943.3 | -2.56 | 0.021481 |
| BF549473 | Ephb2: Eph receptor B2 | -2.51 | 0.011328 |
| AI111605 | Siae: sialic acid acetylesterase | -2.49 | 0.03209 |
| BE119999 | Thap3: THAP domain containing, apoptosis associated protein 3 | -2.46 | 0.005979 |
| NM_013037 | Il1rl1: interleukin 1 receptor-like 1 | -2.45 | 0.037328 |
| AW534159 | Rn.47501.1 | -2.44 | 0.028613 |
| AI230479 | Ulk1: Unc-51 like kinase 1 (C. elegans) | -2.42 | 0.027587 |
| AW527725 | Rn.46636.1 | -2.4 | 0.046563 |
| AI406502 | Rn.16640.1 | -2.38 | 0.011218 |
| BM386775 | Aim1l: absent in melanoma 1-like | -2.37 | 0.021561 |
| U56824 | Klra5 /// LOC502908 /// Ly49s7: killer cell lectin-like receptor, subfamily A, member 5 /// Ly49s8 /// Ly49 stimulatory receptor 7 | -2.37 | 0.03897 |
| AI233361 | Rn.24718.1 | -2.36 | 0.048376 |
| AW916358 | Sptlc1: serine palmitoyltransferase, long chain base subunit 1 | -2.34 | 0.009721 |
| BF397033 | Rnf20: ring finger protein 20 | -2.32 | 0.041243 |
| AA963844 | Fam164a: Family with sequence similarity 164, member A | -2.31 | 0.017332 |
| AI030043 | Rn.17307.1 | -2.31 | 0.02383 |
| AA817920 | Rab39: RAB39, member RAS oncogene family | -2.3 | 0.010509 |
| AA944518 | Rn.34351.1 | -2.28 | 0.017806 |
| AI045487 | Me2: malic enzyme 2, NAD(+)-dependent, mitochondrial | -2.24 | 0.033238 |
| AI103161 | Rn.7614.1 | -2.22 | 0.00745 |
| U04998 | Ptprz1: protein tyrosine phosphatase, receptor-type, Z polypeptide 1 | -2.22 | 0.041829 |
| AA891438 | Pank2: Pantothenate kinase 2 (Hallervorden-Spatz syndrome) | -2.17 | 0.010821 |
| NM_080903 | Trim63: tripartite motif-containing 63 | -2.16 | 0.022565 |
| BI289412 | Aen: apoptosis enhancing nuclease | -2.15 | 0.0399 |
| NM_031123 | Stc1: stanniocalcin 1 | -2.15 | 0.042653 |
| AF311886 | Cyp4v3: cytochrome P450, family 4, subfamily v, polypeptide 3 | -2.13 | 0.006637 |
| BF390621 | Rn.62271.1 | -2.11 | 0.045722 |
| BF524934 | Mrpl12: mitochondrial ribosomal protein L12 | -2.11 | 0.018024 |
| BE105879 | Lrrc4: leucine rich repeat containing 4 | -2.1 | 0.001839 |
| NM_134378 | Sulf1: sulfatase 1 | -2.1 | 0.035737 |
| AI058455 | Rn.19519.1 | -2.08 | 0.049436 |
| AI235545 | Idh1: isocitrate dehydrogenase 1 (NADP+), soluble | -2.08 | 0.016968 |
| BE111095 | Rbm9: RNA binding motif protein 9 | -2.06 | 0.034949 |
| BI296172 | Mfsd3: major facilitator superfamily domain containing 3 | -2.06 | 0.04514 |
| AI059487 | Jph3: junctophilin 3 | -2.05 | 0.029661 |
| BE102644 | Rn.50361.1 | -2.04 | 0.034012 |
| BF387032 | Rn.60197.1 | -2.04 | 0.00262 |
| BI292120 | Baiap2l1: BAI1-associated protein 2-like 1 | -2.04 | 0.039405 |
| L08812 | Tfec: transcription factor EC | -2.04 | 0.007516 |
| NM_133575 | Il1rl2: interleukin 1 receptor-like 2 | -2.04 | 0.017035 |
| BE117105 | Rn.48924.1 | -2.02 | 0.045912 |
| BG376193 | Rn.41732.1 | -2.01 | 0.028839 |
| BG671875 | Fam69b: family with sequence similarity 69, member B | -2.01 | 0.022664 |
| AI711484 | Cc2d1a: coiled-coil and C2 domain containing 1A | 2 | 0.020159 |
| BF558068 | Rn.12460.1 | 2 | 0.007102 |
| AW531976 | Rn.47860.1 | 2.01 | 0.010273 |
| BF409455 | Rn.61362.1 | 2.01 | 0.037238 |
| AI235868 | Rn.42092.1 | 2.02 | 0.002137 |
| BF566738 | Rn.54333.2 | 2.02 | 0.049721 |
| AI168970 | Rn.37723.1 | 2.03 | 0.003151 |
| NM_017296 | Kcnj2: potassium inwardly-rectifying channel, subfamily J, member 2 | 2.03 | 0.013329 |
| AW254078 | Rn.40858.1 | 2.04 | 0.01016 |
| NM_012833 | Abcc2: ATP-binding cassette, sub-family C (CFTR/MRP), member 2 | 2.04 | 0.04458 |
| NM_013074 | Hcrtr2: hypocretin (orexin) receptor 2 | 2.04 | 0.028051 |
| AI235878 | Wnk1: WNK lysine deficient protein kinase 1 | 2.05 | 0.040181 |
| BF391440 | Rn.63077.1 | 2.05 | 0.014577 |
| BF563976 | Irx2: iroquois homeobox 2 | 2.05 | 0.011633 |
| AW251334 | LOC364558: similar to palladin; CGI-151 protein | 2.06 | 0.011362 |
| BI280974 | Rn.78130.1 | 2.06 | 0.024457 |
| L13193 | Foxc2: forkhead box C2 | 2.06 | 0.021086 |
| AI013485 | Rn.24228.1 | 2.07 | 0.029487 |
| AI454552 | Rn.36579.1 | 2.07 | 0.025505 |
| AW144070 | Znf668: zinc finger protein 668 | 2.07 | 0.023766 |
| BF406829 | Rn.64463.1 | 2.07 | 0.004829 |
| BE106653 | Rn.51013.1 | 2.08 | 0.015879 |
| BE106743 | Rn.49089.1 | 2.08 | 0.020004 |
| AI556642 | Rn.38860.1 | 2.09 | 0.00009 |
| BE110066 | Rn.51804.1 | 2.09 | 0.0477 |
| BF408166 | Rn.63425.1 | 2.1 | 0.045214 |
| BI297463 | Rn.66241.1 | 2.1 | 0.004521 |
| BI300942 | Pde8b: phosphodiesterase 8B | 2.1 | 0.041064 |
| BM382879 | Pura: Purine rich element binding protein A | 2.1 | 0.036777 |
| AI502785 | Pcnxl2: pecanex-like 2 (Drosophila) | 2.11 | 0.012337 |
| BG378170 | Rn.66547.1 | 2.11 | 0.010424 |
| NM_012725 | Klkb1: kallikrein B, plasma 1 | 2.11 | 0.011355 |
| AI502924 | Rn.35636.1 | 2.12 | 0.014709 |
| BF288188 | Rn.43267.1 | 2.12 | 0.012662 |
| BF401772 | Rn.59875.1 | 2.12 | 0.033409 |
| BF404508 | Rn.58618.1 | 2.12 | 0.009105 |
| BM390901 | Rn.82556.1 | 2.12 | 0.042698 |
| U07993 | G6pc: glucose-6-phosphatase, catalytic subunit | 2.12 | 0.043643 |
| NM_019344 | Rgs8: regulator of G-protein signaling 8 | 2.13 | 0.008798 |
| BE111497 | Rn.15013.2 | 2.14 | 0.035281 |
| BF410595 | Rn.61753.1 | 2.14 | 0.035593 |
| BG380772 | Lrfn2: leucine rich repeat and fibronectin type III domain containing 2 | 2.14 | 0.049826 |
| BI297375 | LOC688990: Hypothetical protein LOC688990 | 2.14 | 0.004906 |
| AI172451 | Rn.44599.1 | 2.15 | 0.029333 |
| AI233903 | RGD1311103: similar to RIKEN cDNA 2410146L05 | 2.15 | 0.007181 |
| BF393183 | Rn.57999.1 | 2.15 | 0.029955 |
| BF419648 | Cdh20: cadherin 20 | 2.15 | 0.037547 |
| BG379043 | Rn.50315.1 | 2.15 | 0.027345 |
| BF563047 | Rn.46050.2 | 2.16 | 0.015577 |
| AA924452 | Rn.52856.1 | 2.17 | 0.025601 |
| AA923975 | Ptpn4: Protein tyrosine phosphatase, non-receptor type 4 | 2.18 | 0.031005 |
| AI502118 | Rn.18528.1 | 2.18 | 0.009043 |
| AW520939 | Rn.64858.1 | 2.18 | 0.014785 |
| AI169562 | LOC292861: kallikrein | 2.19 | 0.006488 |
| NM_013114 | Selp: selectin, platelet | 2.19 | 0.018075 |
| AA956417 | Rn.23170.1 | 2.2 | 0.036831 |
| AA999128 | Zfp68: Zinc finger protein 68 | 2.2 | 0.035686 |
| AI576825 | Yy1: YY1 transcription factor | 2.2 | 0.02106 |
| BF391635 | Ankrd6: ankyrin repeat domain 6 | 2.2 | 0.02163 |
| BG377504 | Foxi1: forkhead box I1 | 2.2 | 0.00314 |
| BE114146 | Rasip1: Ras interacting protein 1 | 2.21 | 0.00572 |
| BF288281 | Rn.6607.1 | 2.21 | 0.029312 |
| BF416465 | Rn.63303.1 | 2.21 | 0.012103 |
| BG375500 | Rn.42318.1 | 2.21 | 0.019461 |
| NM_021684 | Adcy10: adenylate cyclase 10 (soluble) | 2.21 | 0.023786 |
| D86556 | Pnck: pregnancy upregulated non-ubiquitously expressed CaM kinase | 2.22 | 0.046113 |
| NM_133400 | A1cf: APOBEC1 complementation factor | 2.23 | 0.032672 |
| AA942941 | Rn.43768.1 | 2.24 | 0.048601 |
| BI300335 | Rn.76386.1 | 2.24 | 0.0184 |
| BF393831 | Wdfy3: WD repeat and FYVE domain containing 3 | 2.25 | 0.015596 |
| BG378672 | Metrn: meteorin, glial cell differentiation regulator | 2.25 | 0.041228 |
| BI284578 | Svs6: seminal vesicle secretory protein 6 | 2.25 | 0.000625 |
| BM391441 | Hapln1: hyaluronan and proteoglycan link protein 1 | 2.25 | 0.010492 |
| BF390636 | Aftph: Aftiphilin | 2.27 | 0.010655 |
| BF413103 | Rn.28448.1 | 2.27 | 0.006325 |
| BF523821 | Clca4l: chloride channel calcium activated 4-like | 2.27 | 0.019666 |
| BM387935 | Rn.61103.1 | 2.27 | 0.027913 |
| NM_019304 | Dgkb: diacylglycerol kinase, beta | 2.28 | 0.001459 |
| AI408920 | Rn.43431.1 | 2.29 | 0.018139 |
| AI013040 | March2: membrane-associated ring finger (C3HC4) 2 | 2.3 | 0.038172 |
| AI717493 | Fscn2: fascin homolog 2, actin-bundling protein, retinal (Strongylocentrotus purpuratus) | 2.3 | 0.041024 |
| AI008960 | Hoxa10: homeo box A10 | 2.32 | 0.032371 |
| AI112258 | Epb4.1l1: erythrocyte protein band 4.1-like 1 | 2.32 | 0.043177 |
| AI172180 | Rn.33864.1 | 2.32 | 0.028367 |
| NM_080770 | Scgb2a1 /// Scgb2a2: secretoglobin, family 2A, member 1 /// secretoglobin, family 2A, member 2 | 2.32 | 0.009389 |
| NM_133296 | Slc6a20: solute carrier family 6 (neurotransmitter transporter), member 20 | 2.32 | 0.010386 |
| Y18810 | Grm1: glutamate receptor, metabotropic 1 | 2.32 | 0.000535 |
| AA957487 | Fam183b: family with sequence similarity 183, member B | 2.33 | 0.045229 |
| AI175740 | Alkbh5: alkB, alkylation repair homolog 5 (E. coli) | 2.33 | 0.030497 |
| BE110459 | Slbp: Stem-loop binding protein | 2.33 | 0.003309 |
| NM_133599 | Lgals2: lectin, galactoside-binding, soluble 2 | 2.33 | 0.022817 |
| AA924634 | Rn.15358.1 | 2.34 | 0.01707 |
| BE107308 | Rn.50014.1 | 2.34 | 0.012534 |
| BF412992 | Odf3: outer dense fiber of sperm tails 3 | 2.34 | 0.013053 |
| BI288520 | Ribc2: RIB43A domain with coiled-coils 2 | 2.34 | 0.013023 |
| BE109908 | Rn.51768.1 | 2.37 | 0.012949 |
| AA819053 | Lrrc4c: leucine rich repeat containing 4C | 2.38 | 0.035152 |
| BE101795 | Rn.50127.1 | 2.38 | 0.013135 |
| BF283938 | RGD1562890: RGD1562890 | 2.38 | 0.039918 |
| BI295963 | RGD1305899: similar to Protein C20orf158 | 2.38 | 0.01187 |
| BE108223 | Rn.51430.1 | 2.4 | 0.034971 |
| BF282778 | Rn.55984.1 | 2.4 | 0.025101 |
| BE113790 | Rn.52240.1 | 2.44 | 0.020203 |
| BF543142 | Exosc6: Exosome component 6 | 2.44 | 0.007261 |
| BF396653 | Rn.49014.1 | 2.45 | 0.000343 |
| BF411770 | Rn.61918.1 | 2.45 | 0.03272 |
| NM_022583 | Insl6: insulin-like 6 | 2.45 | 0.043887 |
| AA818585 | Rn.16727.1 | 2.46 | 0.018002 |
| AI044421 | Rn.34381.1 | 2.46 | 0.019559 |
| AI144648 | Tmem16c: transmembrane protein 16C | 2.46 | 0.01998 |
| AA998651 | Rn.7272.1 | 2.47 | 0.018568 |
| AI411724 | Rn.38854.1 | 2.47 | 0.0079 |
| AA875406 | Rn.2907.3 | 2.48 | 0.00423 |
| AB020615 | Prkci: protein kinase C, iota | 2.48 | 0.021273 |
| AI715424 | Rn.41152.1 | 2.5 | 0.022287 |
| BI288159 | Rn.39318.1 | 2.5 | 0.006199 |
| AI059960 | Gba3: glucosidase, beta, acid 3 (cytosolic) | 2.52 | 0.01844 |
| BF405056 | Rn.61701.1 | 2.52 | 0.023876 |
| BF408724 | Rn.62173.1 | 2.54 | 0.02675 |
| BI278347 | Pold3: polymerase (DNA-directed), delta 3, accessory subunit | 2.54 | 0.045864 |
| BF565261 | Meig1: meiosis expressed gene 1 | 2.55 | 0.002937 |
| AA925673 | Fam159b: family with sequence similarity 159, member B | 2.56 | 0.027012 |
| BF386452 | Rn.56274.1 | 2.56 | 0.049738 |
| AI112837 | Foxc2: forkhead box C2 | 2.57 | 0.036477 |
| AI717265 | Rn.41182.1 | 2.57 | 0.042023 |
| BI296648 | Rn.76516.1 | 2.57 | 0.038195 |
| BG374625 | Rn.52840.1 | 2.59 | 0.026708 |
| BE102662 | Rn.62371.1 | 2.6 | 0.003488 |
| BE120039 | Rn.51856.1 | 2.61 | 0.000922 |
| H31734 | Rn.14859.1 | 2.62 | 0.019318 |
| L03202 | Htr6: 5-hydroxytryptamine (serotonin) receptor 6 | 2.62 | 0.026675 |
| NM_017109 | Syn3: synapsin III | 2.62 | 0.013228 |
| AA799992 | RGD1306959: similar to C11orf17 protein | 2.64 | 0.030145 |
| BI289790 | Vgll1: vestigial like 1 (Drosophila) | 2.7 | 0.006823 |
| BI281143 | Lce1c /// Lce1d /// Lce1f /// Lce1s /// RGD1561089: late cornified envelope 1C /// late cornified envelope 1D /// late cornified envelope 1F /// late cornified envelope 1S /// RGD1561089 | 2.73 | 0.00002 |
| AA926196 | Rn.24030.1 | 2.75 | 0.025938 |
| BE116124 | Rn.52624.1 | 2.75 | 0.028097 |
| D10770 | Prkacb: protein kinase, cAMP dependent, catalytic, beta | 2.78 | 0.005356 |
| AB039828 | Mup5: major urinary protein 5 | 2.82 | 0.029901 |
| AI228550 | Rn.12112.1 | 2.82 | 0.021689 |
| AI548146 | Rn.38164.1 | 2.83 | 0.018194 |
| BG667371 | Rn.59653.1 | 2.85 | 0.005456 |
| AI044853 | Rn.16037.1 | 2.86 | 0.02309 |
| AI712398 | Rn.40967.1 | 2.86 | 0.016066 |
| BF415530 | RGD1565726: similar to hypothetical protein A730098P15 | 2.87 | 0.038374 |
| AA818651 | Sipa1l3: signal-induced proliferation-associated 1 like 3 | 2.89 | 0.036921 |
| AI111566 | Rn.21769.1 | 2.95 | 0.030506 |
| BF394624 | Rn.6580.1 | 2.95 | 0.048038 |
| BF564328 | Rn.48323.1 | 2.96 | 0.013524 |
| AI547694 | Rn.43868.1 | 3.03 | 0.018294 |
| AF262319 | Casp9: caspase 9, apoptosis-related cysteine peptidase | 3.06 | 0.022641 |
| AI178991 | Rn.35551.1 | 3.07 | 0.007759 |
| BG381453 | Eif4g1 /// LOC679630: eukaryotic translation initiation factor 4 gamma, 1 /// similar to eukaryotic translation initiation factor 4, gamma 1 isoform a | 3.1 | 0.043127 |
| NM_053836 | Il2: interleukin 2 | 3.11 | 0.008794 |
| BM384729 | Lao1: L-amino acid oxidase 1 | 3.12 | 0.029728 |
| AA859029 | Fabp12: Fatty acid binding protein 12 | 3.14 | 0.022514 |
| BF416438 | Myrip: myosin VIIA and Rab interacting protein | 3.15 | 0.03245 |
| AI112506 | Rn.26795.1 | 3.2 | 0.030311 |
| BF394303 | Rn.61309.1 | 3.22 | 0.01425 |
| BI297081 | Rn.52414.1 | 3.24 | 0.030849 |
| BF291108 | Rn.56036.1 | 3.27 | 0.005581 |
| BF399642 | Rn.59419.1 | 3.28 | 0.046485 |
| AA899105 | Rn.32370.1 | 3.3 | 0.004684 |
| U36899 | Vnr2: vomeronasal receptor 2 | 3.3 | 0.044127 |
| AI071992 | Rn.43541.1 | 3.44 | 0.012719 |
| BI284367 | Rn.54114.1 | 3.5 | 0.026887 |
| AI576663 | Rn.39390.1 | 3.51 | 0.008575 |
| BF403919 | Rn.63591.1 | 3.52 | 0.006555 |
| BG378431 | Rn.73208.1 | 3.57 | 0.026104 |
| AI555202 | Nin: ninein (GSK3B interacting protein) | 3.58 | 0.007809 |
| AI575616 | Rn.26735.1 | 3.58 | 0.008428 |
| BF392912 | Rn.63404.1 | 3.65 | 0.011581 |
| BF391815 | Rn.62931.1 | 3.66 | 0.022987 |
| BE120031 | Rn.53440.1 | 3.7 | 0.039096 |
| AI072348 | MAST1: microtubule associated serine/threonine kinase 1 | 3.82 | 0.016602 |
| BF401689 | Rn.61205.1 | 3.87 | 0.027137 |
| BF523421 | Zfp90: zinc finger protein 90 | 4.11 | 0.001809 |
| BF411781 | Filip1: filamin A interacting protein 1 | 4.14 | 0.010763 |
| BF565954 | Rn.49106.1 | 4.16 | 0.047158 |
| BF408103 | Rn.64327.1 | 4.24 | 0.008401 |
| AI411908 | Rn.43643.1 | 4.25 | 0.008685 |
| BE107074 | Rn.50650.1 | 4.25 | 0.015111 |
| AW915163 | Rn.7095.1 | 4.28 | 0.018929 |
| BE109825 | Parvb: parvin, beta | 4.47 | 0.029519 |
| BF417978 | Rn.63329.1 | 4.76 | 0.019789 |
| BM385230 | Mttp: microsomal triglyceride transfer protein | 4.78 | 0.028647 |
| AA962978 | Fam149a: family with sequence similarity 149, member A | 5.07 | 0.000356 |
| AI070110 | C1ql1: complement component 1, q subcomponent-like 1 | 5.48 | 0.048946 |
| BF420102 | Dnajc7: DnaJ (Hsp40) homolog, subfamily C, member 7 | 5.68 | 0.021715 |
| BE107070 | Rn.53920.1 | 5.93 | 0.009733 |
| AW527736 | RGD1562107: similar to class-alpha glutathione S-transferase | 5.99 | 0.010918 |
| AA946199 | Snai1: snail homolog 1 (Drosophila) | 6.18 | 0.000504 |
| BE109649 | Rnf207: ring finger protein 207 | 6.72 | 0.029649 |
| BI292168 | Hoxb13: homeo box B13 | 6.8 | 0.004039 |
| BF413240 | Rn.46481.1 | 6.9 | 0.038911 |

**Supplemental Table S2.** Genes Ontology (GO) analysis of the microarray data*

| GO Categories | Rank/ID | Term | List Hits | P-value |
| --- | --- | --- | --- | --- |
| Biological Process | 1. GO:0006468 2. GO:0007243 3. GO:0006974 4. GO:0048593 5. GO:0016310 6. GO:0042462 7. GO:0071456 8. GO:0006836 9. GO:0060707 10. GO:0007205 11. GO:0070327 12. GO:0001567 | Protein phosphorylation  Intracellular protein kinase cascade  Response to DNA damage stimulus  Camera-type eye morphogenesis  Phosphorylation  Eye photoreceptor cell development  Cellular response to hypoxia  Neurotransmitter transport  Trophoblast giant cell differentiation  Activation of protein kinase C activity by G-protein coupled receptor protein signaling pathway  Thyroid hormone transport  Patterning of blood vessels | 9  3  3  3  3  3  3  2  2  2  2  2 | 1.69e-02  1.67e-02  3.5e-02  1.95e-04  3.17e-02  1.62e-04  2.18e-03  3.47e-02  1.26e-03  2e-02  6.79e-04  2e-02 |
| Molecular Function | 1. GO:0000287 2. GO:0008134 3. GO:0004672 4. GO:0005215 5. GO:0003690 6. GO:0001948 7. GO:0017022 8. GO:0030674 9. GO:0048306 10. GO:0003729 11. GO:0004888 12. GO:0004908 | Magnesium ion binding  Transcription factor binding  Protein kinase activity  Transporter activity  Double-stranded DNA binding  Glycoprotein binding  Myosin binding  Protein binding, bridging  Calcium-dependent protein binding  mRNA binding  Transmembrane receptor activity  Interleukin-1 receptor activity | 5  5  4  4  3  2  2  2  2  2  2  2 | 5.83-03  1.52e-02  3.39e-02  4.87e-02  4.41e-02  3.32e-02  5.59e-03  2.51e-02  2.38e-02  4.37e-02  3.75e-02  2.61e-04 |
| Cellular Component | 1. GO:0005634 2. GO:0005886 3. GO:0005624 4. GO:0005625 5. GO:0043025 6. GO:0016324 7. GO:0045202 8. GO:0030425 9. GO:0005667 10. GO:0030424 11. GO:0030672 12. GO:0005814 | Nucleus  Plasma membrane  Membrane fraction  Soluble fraction  Neuronal cell body  Apical plasma membrane  Synapse  Dendrite  Transcription factor complex  Axon  Synaptic vesicle membrane  Centriole | 34  25  8  8  7  6  6  6  5  4  2  2 | 4.8e-02  1.57e-02  1.47e-02  4.15e-03  5.07e-03  3.41e-03  1.24e-02  6.96e-03  1.27e-03  4.22e-02  4.15e-02  2.64e-02 |

*The top 12 includes cellular component, molecular function and Biological process, describes differentially expressed genes function from microarray by DHI treatment. ID, ID entry in GO database; Term, differently expressed; List Hits, the number of genes annotated by GO Biological process, molecular function and cellular component category or annotation cluster within the analyzed list of target genes; P-value, the significance p-value of the gene enrichment of the considered GO Biological process, molecular function and cellular component category or annotation cluster, calculated with a unilateral Fisher exact test.

**Supplemental Table S3. Kyoto Encyclopedia of Genes and Genomes (KEGG) classification of the microarray data***

| **ID** | **Term** | **List Hits** | **P-value** | **Genes ID’s** | **Gene Names** |
| --- | --- | --- | --- | --- | --- |
| 04910 | Insulin signaling pathway | 4 | 5.35e-03 | 24701  25634  293508  84006 | Pygm  G6pc  Prkacb  Prkci |
| 00500 | Starch and sucrose | 3 | 1.97e-0.3 | 24701  25634  289687 | Pygm  G6pc  Gba3 |
| 00480 | Glutathione metabolism | 2 | 3.61e-0.2 | 24479  363205 | Idh1  RGD1562107 |
| 04150 | mTOR signaling pathway | 2 | 3.15e-0.2 | 360827  83785 | Ulk1  Vegfa |
| 00460 | Cyanoamino acid metabolism | 1 | 3.70e-0.2 | 289687 | Gba3 |
| 00400 | Phenylalanine, tyrosine and tryptophan biosynthesis | 1 | 3.18e-0.2 | 298483 | Lao1 |

***** Differentially expressed genes were imported to the KEGG database and 6 pathways were found significantly enriched with the differentially expressed genes according to the Fisher’s exact test, p-values are considered to be impacted by the DHI treatment. ID, ID entry in KEGG database; Term, KEGG pathway; List Hits, the number of genes annotated by the considered KEGG category or annotation cluster within the analyzed list of target genes; P-value, the significant p-value of the gene enrichment of the considered KEGG category or annotation cluster, calculated with a unilateral Fisher exact test; Genes ID’s, ID of differentially expressed genes; Gene names, Gene names of differentially expressed genes.

**Supplemental Table S4. Forward and reverse primers of selected genes are from differentially expressed genes of microarray data*.**

| Genes | Oligo names | Sequences (5’ to 3’) |
| --- | --- | --- |
| Kallikrein | qRat Kallikrein fwd | ATGCAGTGAGCCCCATAACC |
|  | qRat Kallikrein rev | CAGAGTTTGCCGTCCCTCAT |
| Klkb1 | qRat Klkb1 fwd | TGTAAGCCATGTCGTCACCC |
|  | qRat Klkb1 rev | CGTCTCCCACTCATTCGTGT |
| Kcnj2 | qRat Kcnj2 fwd | GACGCCTTCATCATTGGTGC |
|  | qRat Kcnj2 rev | TGTGACACTCAGGGGTTTTCC |
| Htr6 | qRat Htr6 fwd | AGCTCACAGCCCAGCTTCTGCT |
|  | qRat Htr6 rev | GTATCTCAGGCTCCACAGAGTCTG |
| vWF | qRat vWF fwd | CCTACACTTTGTGGATGTGGATGAC |
|  | qRat vWF rev | GCGGAAGCCATTGGACAGA |
| B_2_M | qRat B_2_M fwd | CGTGATCTTTCTGGTGCTTGTC |
|  | qRat B_2_M rev | TTCTGAATGGCAAGCACGAC |

*Klkb1: kallikrein B, plasma 1; Kcnj2: potassium inwardly-rectifying channel, subfamily J, member 2; Htr6: 5-hydroxytryptamine (serotonin) receptor 6; Vwf: von Willebrand factor; B_2_M, beta-2-microgrobulin; fwd, forward; rev, reverse.

**References**

1. Patterson TA, Lobenhofer EK, Fulmer-Smentek SB, Collins PJ, Chu TM, Bao W, et al. Performance comparison of one-color and two-color platforms within the microarray quality control (maqc) project. *Nature biotechnology*. 2006;24:1140-1150

2. Mane SP, Evans C, Cooper KL, Crasta OR, Folkerts O, Hutchison SK, et al. Transcriptome sequencing of the microarray quality control (maqc) rna reference samples using next generation sequencing. *BMC Genomics*. 2009;10:264

3. Shi J, Li M, Jiao Z, Zhang J, Feng Y, Shao B. Microarray analysis of gene expression in mouse (strain 129) embryonic stem cells after typical synthetic musk exposure. *Bulletin of environmental contamination and toxicology*. 2013;90:17-21

4. Zhu YC, Guo Z, He Y, Luttrell R. Microarray analysis of gene regulations and potential association with acephate-resistance and fitness cost in lygus lineolaris. *PLoS One*. 2012;7:e37586

5. Deng J, Calvert V, Pierobon M. Microarray data analysis: Comparing two population means. *Methods Mol Biol*. 2012;823:325-346
